# Assessing the Role of Polyamine Metabolites in Blood and the DNA Methylation of *Mycobacterium Tuberculosis* in Patients with Multidrug-Resistant Tuberculosis

**DOI:** 10.7150/ijms.102568

**Published:** 2025-03-10

**Authors:** Li Yan, Xinxin Niu, Kuidi Liang, Feifeng Guan, Xiaolin Yu, Ziyu Ye, Mingyuan Huang, Hancheng Liang, Xinguang Zhong, Jincheng Zeng

**Affiliations:** 1 Dongguan Key Laboratory of Tuberculosis Prevention and Control, Dongguan Sixth People's Hospital, Dongguan 523008, Guangdong, China.; 2 Dongguan Key Laboratory of Medical Bioactive Molecular Developmental and Translational Research, Guangdong Provincial Key Laboratory of Medical Immunology and Molecular Diagnostics, Guangdong Medical University, Dongguan 523808, Guangdong, China.; 3 Xinghai Institute of Cell, Guangdong Xianhua Institute for Medical Research, Dongguan 523808, Guangdong, China.

**Keywords:** Tuberculosis, Drug-resistant tuberculosis, Polyamine, DNA methylation.

## Abstract

**Background:** Tuberculosis (TB) is the second largest infectious disease killer in China, and the increasing prevalence of drug-resistant TB patients complicates treatment efforts and raises associated costs. Research on the mechanisms and characteristics of drug-resistant TB contributes to the discovery of new drug targets and the development of new anti-tuberculosis drugs.

**Methods:** In this study, high-performance liquid chromatography (HPLC) was used to detect the content of polyamine metabolites, while western blotting, qPCR and ELISA were used to detect the expression of polyamine metabolism-related enzymes. The Oxford Nanopore Technologies (ONT) sequencing was applied to profile DNA methylation in multidrug-resistant *Mycobacterium tuberculosis* (*Mtb*). Gene ontology (GO) analysis and Kyoto Encyclopedia of Genes and Genomes (KEGG) pathway enrichment analysis were performed on the screened differentially hypermethylated genes. Furthermore, STRING and Cytoscape software were used to construct a protein-protein interaction (PPI) network to identify the key genes.

**Results:** The findings indicated the spermidine (SPD) and polyamine metabolism-related enzymes were elevated in the peripheral blood of TB patients. In addition, the production of polyamines and polyamine metabolism-related enzymes was increased in the peripheral blood of multidrug-resistant tuberculosis (MDR-TB) patients. GO and KEGG analyses showed that the differentially hypermethylated genes were mainly enriched in arginine metabolism. The PPI network analysis identified the top five key genes with the highest degrees: *moaX*, *vapC49*, *vapB49*, *highA3* and* nuoC*.

**Conclusions:** Polyamine metabolites were increased in the peripheral blood of MDR-TB patients. The differentially hypermethylated genes in multidrug-resistant *Mtb* are involved in the arginine biosynthetic process, the differentially methylated genes may play an important biological role in the multidrug resistance of *Mtb*.

## Introduction

Tuberculosis (TB), a chronic infectious disease caused by *Mycobacterium tuberculosis* (*Mtb*) infection 1, is one of the global public health problems 2 and a major cause of death throughout the world 3, 4. Despite the availability of effective vaccines and treatments, tuberculosis remains a difficult disease to overcome 5. Currently, the prevention and treatment of tuberculosis have many dilemmas, primarily in the following aspects: firstly, there are problems with the diagnostic tools for tuberculosis, such as poor specificity, time consumption, and low sensitivity 6, which lead to a delay in the treatment of many tuberculosis patients who are unable to be diagnosed in a timely and accurate manner. In addition, the existing diagnostic methods have difficulties in distinguishing the active tuberculosis infection (ATB) and latent tuberculosis infection (LTBI), and this makes it impossible for high-risk populations to be screened out, thus increasing the risk for healthy individuals to be exposed to the infections 7-9. Secondly, the emergence of multidrug-resistant tuberculosis (MDR-TB) and the increase in the difficulty of its treatment are also the major challenges currently faced by the prevention and treatment of TB. The emergence of drug-resistant TB strains such as MDR-TB and extensively drug-resistant tuberculosis (XDR-TB) has dramatically reduced the effectiveness of antituberculosis treatment and increased the difficulty and cost of treatment 10. Finally, a better knowledge of the immune mechanisms and the immune microenvironment of TB is essential for TB prevention and treatment. Since *Mtb* can remain dormant in the host, these dormant *Mtb* have the risk of impacting the disease progression by modulating the host's immune cells, as well as the risk of being reactivated 11. Currently, our understanding of the immune mechanisms and immune microenvironment of TB is inadequate, which limits our development of a more effective diagnostic and therapeutic approach. Meanwhile, the effectiveness of prevention initiatives is inadequate since we do not yet have a thorough understanding of the pathogenesis of TB. Therefore, in order to achieve the goal of eliminating TB by 2035 12, 13, we need to strengthen our research in TB immune mechanisms and immune microenvironment, develop more accurate and rapid diagnostic methods, search for new therapeutic targets, as well as formulate more effective preventive measures 14. It is also essential to reinforce international cooperation and communication, as overcoming this global public health challenge is achievable only through the collaborative efforts of researchers and institutions.

The burden of TB in China remains severe, particularly the prevalence of MDR-TB and HIV co-infection 15, which makes the situation of TB control even more challenging, as TB has become the No.1 health-threatening killer among all infectious diseases 16. Due to the absence of an effective surveillance tool 17, the cure rate is relatively low in the case of multidrug-resistant tuberculosis (MDR-TB). Therefore, it is critical to comprehend the underlying molecular mechanisms of MDR-TB and to seek new biomarkers for the prevention and treatment of MDR-TB 18. Multidrug-resistant tuberculosis is categorized into primary multidrug-resistant tuberculosis and acquired multidrug-resistant tuberculosis according to the different pathogenesis. Primary MDR-TB is caused by transmission of multidrug-resistant *Mtb* and represents a relatively high proportion of MDR-TB, whereas acquired MDR-TB occurs during the treatment process, which is currently the main source of MDR-TB. Therefore, it is highly significant to elucidate the acquisition and transmission of *Mtb* multidrug resistance, which can be an effective way to control multidrug-resistant tuberculosis 19.

Polyamines, metabolically generated from arginine, are essential polymeric cations in all cells 20 which play a crucial regulatory role in thyroid and nucleic acid metabolism 21. Polyamines are a group of small molecule compounds that are commonly found in various cells with biological activities, mainly including spermidine (SPD) and spermine (SPM) 22, several studies have demonstrated that they are associated with a variety of immune-regulatory functions and diseases 23 and perform an important physiological role in the body 24. It has also been reported that polyamines can induce anti-inflammatory macrophage polarization, regulate the inflammatory response, decrease the expression of corresponding genes in macrophages, and attenuate the production of inflammatory mediators 25, 26. Furthermore, polyamines can also affect the metabolism and function of natural killer (NK) cells, enhancing the differentiation of regulatory T cells (Tregs) and improving the function of B lymphocytes 27, 28. The balance between these cells and their subpopulations has a certain impact on the acquisition and progression of TB and is strongly associated with disease prognosis. Polyamines perform important physiological functions in the living cells, including regulation of ion channels, transcription and translation of genes, and antioxidant damage. Hirsch *et al.* isolated spermine from extracts of tissue in acidified dilute ethanol and found that it was able to inhibit the growth of a wide range of mycobacteria, and that this inhibitory substance had the same activity against different types of MTB 29. Emani *et al.* first demonstrated that spermine enhances the activity of anti-tuberculosis drugs, which provided a new direction and basis for subsequent studies 30.

However, the functions and mechanisms of polyamines in the development of multidrug-resistant tuberculosis and in the DNA methylation of multidrug-resistant* Mtb* are still unclear. The aim of this study is to further characterize the polyamine content of the peripheral blood of MDR-TB patients and the level of DNA methylation of multidrug-resistant *Mtb*.

## Materials and Methods

### Subjects

Blood samples from healthy volunteers (n=48) and TB patients (n=60) were provided by Dongguan Sixth People's Hospital. Inclusion criteria were being aged 17 to 76 years; having at least 1 positive sputum smear result or a positive culture of Mycobacterium tuberculosis; having tuberculosis lesions on chest imaging; or having tuberculosis pathology confirmed by tissue biopsy. Exclusion criteria were having other lung diseases (such as pneumonia, lung cancer, bronchiectasis, lung abscess, or chronic obstructive pulmonary disease); having other febrile diseases (such as typhoid fever, leukemia, or sepsis); or having other major lesions. Blood samples from healthy volunteers (HV) collected during the same period were selected as the control group. The basic information of the participants is summarized in **Table 1** below. This study was approved by the ethics committees of the Dongguan Sixth People's Hospital (Approval No. Z2022-007). Informed consent was obtained from all participants.

### Reagents

Sodium hydroxide (NaOH), sodium bicarbonate (NaHCO_3_), ammonia (analytical reagent), perchloric acid (HClO_4_; analytical reagent), chloroform (CHCl_3_; analytical reagent) and isopropyl alcohol were purchased from Damao chemical reagent factory. Acetonitrile (C2H3N; HPLC) was purchased from Tianjin Kemiou chemical reagent Co.,Ltd. Dansyl chloride (DNSCl) was purchased from Shanghai Macklin biochemical technology Co., Ltd. Spermine (standard), spermidine (standard) and dimethyl sulfoxide (DMSO) were purchased from Sigma-Aldrich. Spermidine/Spermine N1-Actyltransferase (SSAT) ELISA kit, hypoxia-inducible factor-1α (HIF-1α) ELISA kit and nitric oxide synthase (NOS) ELISA Kit were purchased from Quanzhou Ruixin biotechnology Co.,Ltd. PBMCs isolation medium was purchased from Tianjin Haoyang biological manufacture Co., Ltd. Spermine oxidase (SMOX) antibody, ornithine decarboxylase 1(ODC1) antibody, spermidine synthase (SRM) antibody and GAPDH antibody were purchased from Proteintech Group. RIPA lysis buffer, Proteinase K, BCA protein assay kit, enhanced chemiluminescence (ECL) detection reagent, HRP-labeled goat anti-mouse IgG and HRP-labeled goat anti-rabbit IgG were purchased from Beyotime biotechnology. TRIzol was purchased from Thermo Fisher Scientific. *Evo M-MLV* reverse transcription kit was purchased from Hunan Accurate biotechnology. TB Green^®^ Premix Ex Taq™ was purchased from Takara.

### High performance liquid chromatography (HPLC)

The levels of spermine and spermidine in the serum were determined by high performance liquid chromatography (HPLC). A total of 250 μL of peripheral blood was collected and subjected to centrifugation, resulting in the addition of 200 μL of the supernatant to 300 μL of cold 5% HClO_4_, followed by further centrifugation to obtain 400 μL of supernatant. To successfully prepare the derivative, 200 μL of NaOH and 400 μL of saturated NaHCO_3_ solution were added to the standards and samples (400 μL), respectively, after which 400 μL of dansyl chloride (DNSCl) was added to the mixture. The reaction was allowed to proceed for 45 mins in the dark and was subsequently terminated by the addition of 100 μL strong ammonia. The reaction mixture was then extracted by adding 800 μL of trichloromethane and centrifuged at 3000 r/min for 10 mins; 700 μL of the lower organic phase was dried under nitrogen and redissolved in 500 μL of acetonitrile to yield the sample solution. Finally, the sample solution was filtered through a 0.45 μm filter membrane and analyzed using HPLC with a C18 column (4.6 mm × 250 mm, 5 μm). Mobile phase: phase A was ultrapure water, phase B was acetonitrile, gradient elution (**Table 2**); fluorescence detector: excitation wavelength is 340 nm, emission wavelength is 510 nm; flow rate: 1 mL/min; injection volume: 10 μL; column temperature: 40 °C.

### Isolation of PBMCs

In order to isolate peripheral blood mononuclear cells (PBMCs), 5 mL of peripheral whole blood was initially loaded onto PBMCs isolation medium and centrifuged at 600 g for 30 mins. Then we used a Pasteur pipette to carefully aspirate the mononuclear cell layer and added it to 10 mL of PBS and mixed well by pipetting up and down. After centrifugation at 250 g for 10 mins, the supernatant was discarded, and 5 mL of PBS was used to resuspend the cells. We repeated the centrifugation for another 10 mins. Then, we discarded the supernatant and added 1 mL of cell cryopreservation solution to the pellet in order to resuspend the cells, and we transferred the cell suspension to a 2 mL cell cryovials. Cryovials were promptly transferred to -80 °C freezers for step-down freezing at -1 °C/min in a freezing container (Thermo Fisher Scientific) for at least 12 h before moving to liquid nitrogen for long-term storage.

### Quantitative real-time PCR (qPCR)

Total RNA was extracted from cells by using TRIzol reagent. Nanodrop 2000 was used to determine the RNA concentration. Reverse transcription was performed with 1 μg of RNA in a thermocycler (Zhuhai Hema medical instrument Co., LTD), using a *Evo M-MLV* reverse transcription kit. qPCR was carried out using a Real-Time PCR System supplied by Hangzhou Bioer technology Co., LTD and using TB Green^®^ Premix Ex Taq™. *GAPDH* was used as a control. Relative expressions were calculated using the comparative threshold cycle (2^-ΔΔCt^) method. The primer sequence is shown in **Table 3** below.

### Western blotting (WB)

The collected PBMCs were resuspended on ice with RIPA lysis buffer and then ultrasonically lysed for 30 s. The cell lysate was centrifuged at 12000 rpm/4 °C for 10 mins, and the supernatant was collected for further analysis. Total protein concentration of cell lysates was determined using the BCA Protein Assay Kit according to the manufacturer's instruction. Briefly, standard solutions and protein samples were added to separate wells of a 96-well microplate, after which BCA working reagent was added to each well. The plate was incubated at 37°C for 30 mins, the absorbance at 562 nm was measured using a microplate reader. Protein samples were separated by SDS-PAGE and transferred onto 0.22 μm PVDF membranes, the membranes were incubated with primary and HRP-conjugated secondary antibodies. Finally, the protein bands on the membranes were visualized using ECL detection reagent and imaged with the Azure Imaging System (Azure Biosystems). The integrated density of the bands was quantitatively analyzed using ImageJ software, with the band of GAPDH serving as an internal loading control.

### Enzyme-linked immunosorbent assays

The concentrations of spermidine/spermine N1-actyltransferase (SSAT), HIF-1α and nitric oxide synthase (NOS) in serum was detected using ELISA kits according to the manufacturer's instructions. The blood specimens were centrifuged at 4000 rpm for 20 mins. The supernatant was detected by ELISA analysis. Briefly, fifty microliters of standards and samples were added to their respective wells. Subsequently, horseradish peroxidase (HRP)-labeled antibody was added to the wells. Then reaction plate was then covered with an adhesive film and incubated in a 37 °C incubator for 60 mins in the dark. Following incubation, the plate was washed 5 times with 200 μL wash buffer per well. Next, 50 μL of substrate A and B were added to each well, and the mixture was incubated at 37 °C for 15 mins. Lastly, 50 μL of the termination solution was added to each well and the OD value at 450 nm of each well was measured within 15 mins.

### DNA extraction from bacteria

Three sputum samples were collected from drug-sensitive tuberculosis patients and three sputum samples from multidrug-resistant tuberculosis patients at Dongguan Sixth People's Hospital. The sputum samples were mixed with 1 mL of protective solution and then centrifuged at 12,000 rpm for 15 mins to discard the supernatant. The bacteria were obtained and flash frozen in liquid nitrogen, followed by lysis with an SDS solution containing proteinase K. After lysis, the samples were cooled to room temperature and then centrifuged. The supernatant was added to a mixture of chloroform and isoamyl alcohol (24:1) and extracted twice; subsequently, the DNA was precipitated using isopropanol, mixed gently by inversion before centrifugation. Following the removal of the waste liquid, the DNA precipitate was washed with 75% ethanol. After drying the DNA precipitate, elution buffer was added, and the sample was digested with RNA enzymes before being purified with a purification column. The DNA underwent further purification using Ampure XP beads and was analyzed for quality control using Nanodrop, Qubit and electrophoresis.

### Whole-genome DNA methylation sequencing

Oxford Nanopore Technologies (ONT) sequencing was employed to profile DNA methylation in *Mtb*. Three DNA samples from drug-sensitive *Mtb* and three DNA samples from multidrug-resistant *Mtb* were selected for purification. The library construction and sequencing were performed by Shenzhen E-GENE Co.,Ltd. Following sequencing, the differentially methylated genes were subjected to Gene ontology (GO) and Kyoto Encyclopedia of Genes and Genomes (KEGG) functional enrichment analysis, and the protein-protein interaction (PPI) network diagram was constructed using String and Cytoscape software.

### Statistical analysis

All numerical data are presented as mean ± standard error of the mean (SEM). Differences between the two groups were analyzed by Student's t-test using GraphPad Prism 9.0 software (USA). A *P*-value of less than 0.05 was considered statistically significant. All experiments were performed in triplicate.

## Results

### The metabolic level of spermidine and the proteomic level of polyamine metabolism-related enzymes were elevated in the peripheral blood of tuberculosis patients

**Figure 1:**
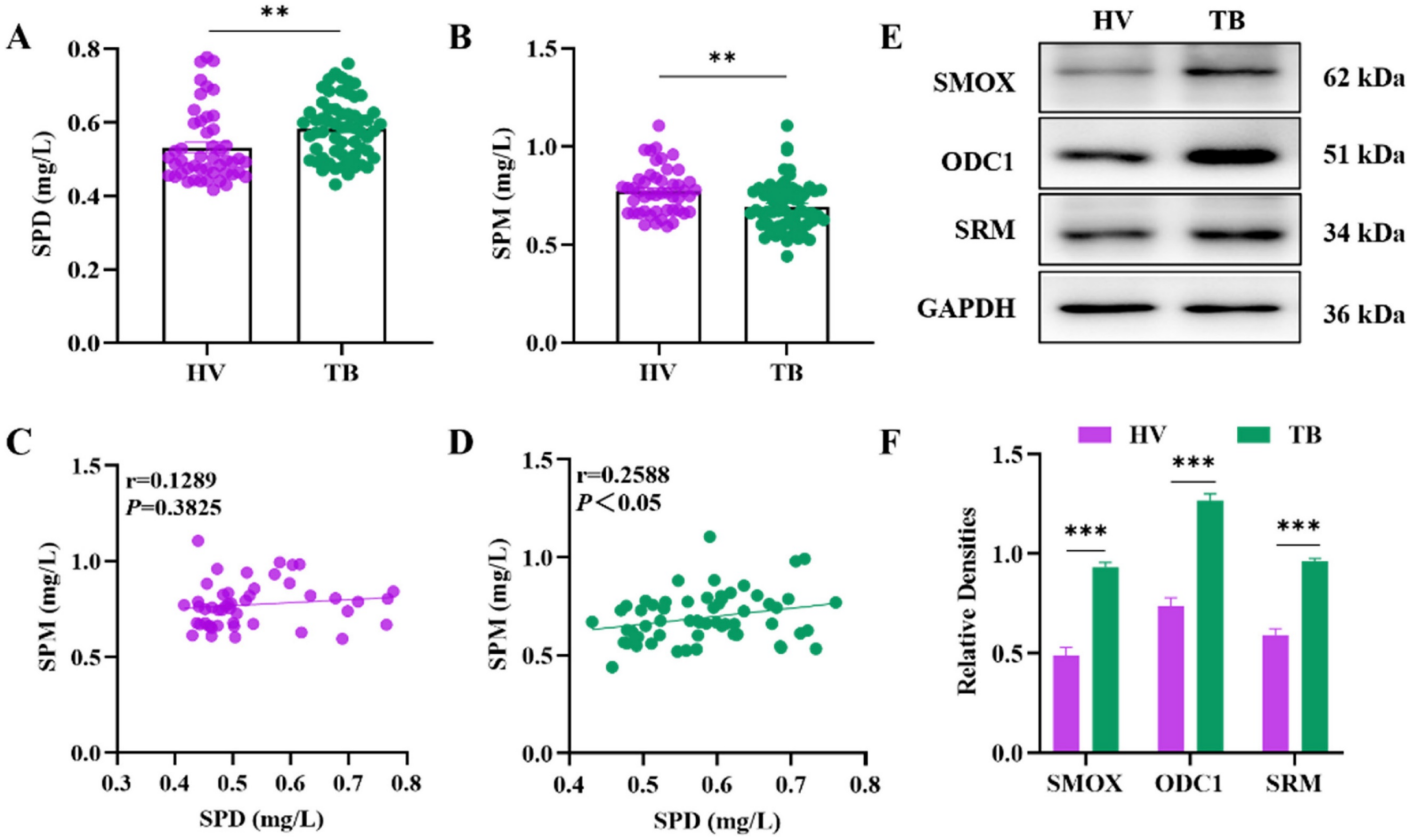
Analysis of the levels of peripheral blood spermidine, spermine and polyamine metabolism-related enzymes in the HV and TB patients. (**A**) and (**B**) Concentrations of SPD and SPM by using HPLC in peripheral blood. (**C**) Correlation analysis between SPD and SPM levels in the HV. (**D**) Correlation analysis between SPD and SPM levels in TB patients. (**E**) The protein production levels of SMOX, ODC1 and SRM in HV and TB patients were detected by western blot. (**F**) Representative semi-quantification of protein band densities normalized to GAPDH from the western blot analysis. **P < 0.01, and ***P < 0.0001.

Utilizing HPLC to detect the content of peripheral blood polyamines among HV and TB patients, the results showed that the level of spermidine increased and spermine decreased in TB patients compared with that of HV (**Figure 1A, 1B**), and that there was a positive correlation between the levels of spermidine and spermine in TB patients (**Figure 1D**), which was statistically significant (*P*<0.05). Polyamines are produced by the metabolism of arginine, and their synthesis is mainly regulated by SMOX (spermine oxidase), ODC1 (ornithine decarboxylase 1) and SRM (spermidine synthase). In this study, the productions of SMOX, ODC1 and SRM protein were evaluated using WB and further quantified. The results showed an increased protein expression of SMOX, ODC1 and SRM in TB patients, which was statistically significant by quantitative analyses (**Figure 1E, 1F**).

### The metabolic level of polyamines and production level of polyamine metabolism-related enzymes were elevated in the peripheral blood of MDR-TB patients

**Figure 2:**
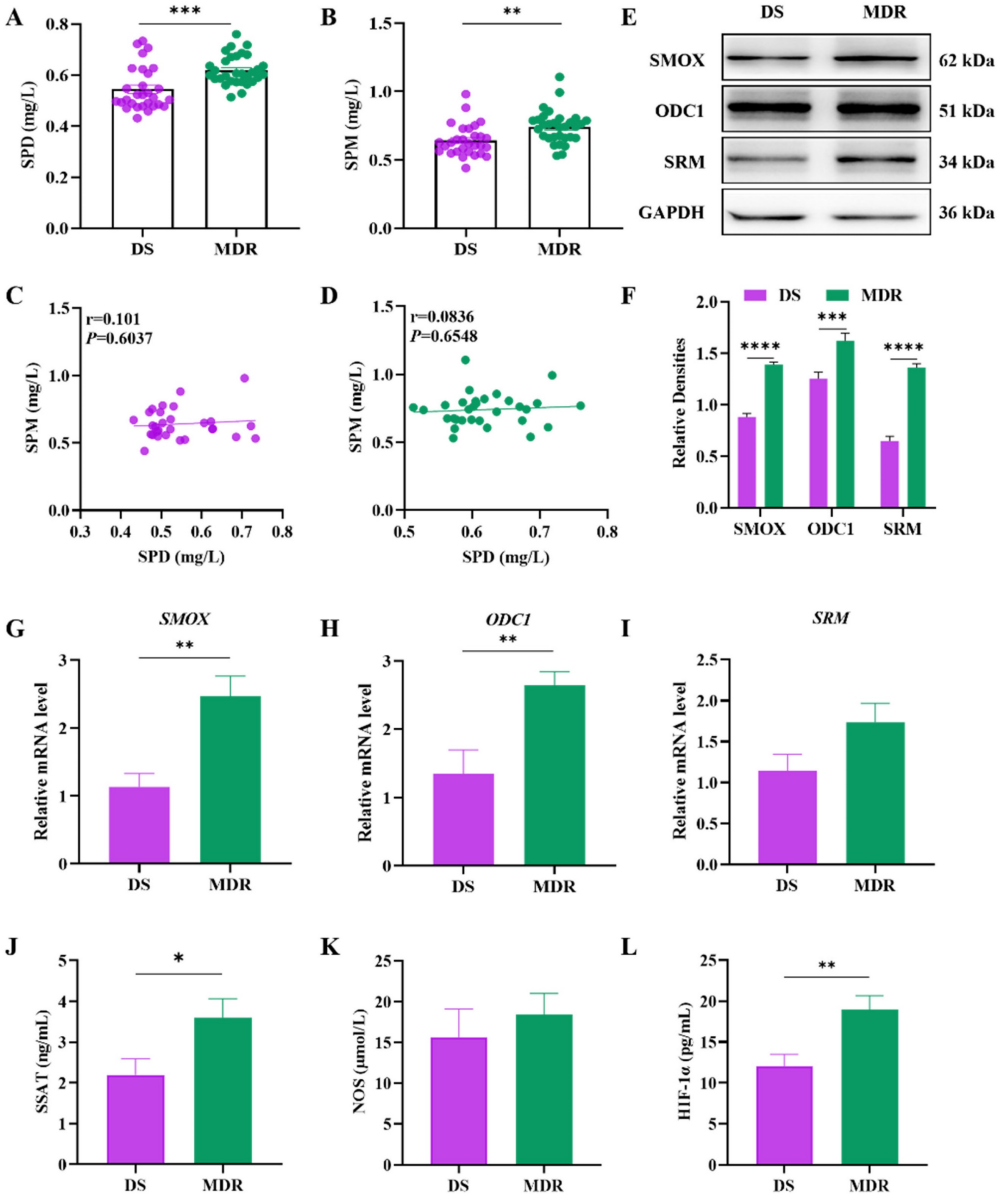
Analysis of the levels of spermidine and spermine and related metabolic enzymes in the peripheral blood of the DS-TB patient group and the MDR-TB patient group. (**A-B**) Concentrations of SPD and SPM by HPLC in peripheral blood. (**C**) Correlation analysis between SPD and SPM levels in the DS-TB patient group. (**D**) Correlation analysis between SPD and SPM levels in MDR-TB patients. (**E**) Protein expression levels of SMOX, ODC1 and SRM by western blot. (**F**) Representative semi-quantification of protein band densities normalized to GAPDH from the western blot analysis. (**G**-**I**) The relative mRNA expression of SMOX, ODC1 and SRM by qPCR. (**J-L**) The levels of SSAT, HIF-1α and NOS in peripheral blood by ELISA. *P < 0.05, **P < 0.01, ***P < 0.001, and ****P < 0.0001.

Utilizing HPLC to detect the polyamine levels of the peripheral blood of MDR-TB and DS-TB patients, we found statistically significant elevated levels of spermidine and spermine among MDR-TB patients (**Figure 2A, 2B**). The expression of polyamine metabolism-related enzymes, including SMOX, ODC1 and SRM, was detected by WB and qPCR experiments, and the results showed an increase in both protein and mRNA expression of SMOX, ODC1 and SRM in MDR-TB patients compared to the DS-TB group, with statistical significance by the quantitative analysis (**Figure 2E-I**). SSAT serves a key enzyme in the catabolism of polyamines, and it takes an important role in the maintenance of the balance of polyamine metabolism *in vivo*. HIF-1α is up-regulated by AMPK (Adenosine 5'-monophosphate (AMP)-activated protein kinase) activation and mitochondrial reactive oxygen species (mtROS) and is necessary for the expression of anti-inflammatory genes and the induction of autophagy. In this study, we analyzed the levels of SSAT, HIF-1α and NOS by ELISA. The results demonstrated that the levels of SSAT and HIF-1α in the peripheral blood of MDR-TB patients were elevated (**Figure 2J, 2L**), while there was no significant difference in the content of NOS compared to those of DS-TB patients (**Figure 2K**).

### GO and KEGG analyses showed that the hypermethylated genes were mainly enriched in arginine metabolism

Next, we employed Oxford Nanopore Technologies (ONT) sequencing to profile DNA methylation in multidrug-resistant* Mycobacterium tuberculosis*. We selected six patients with both clinically and microbiologically confirmed TB, comprising three cases of drug-sensitive TB and three cases of multidrug-resistant TB. Sputum samples were collected from these six patients, from which *Mycobacterium tuberculosis* was isolated, and DNA was extracted for methylation sequencing.

**Figure 3:**
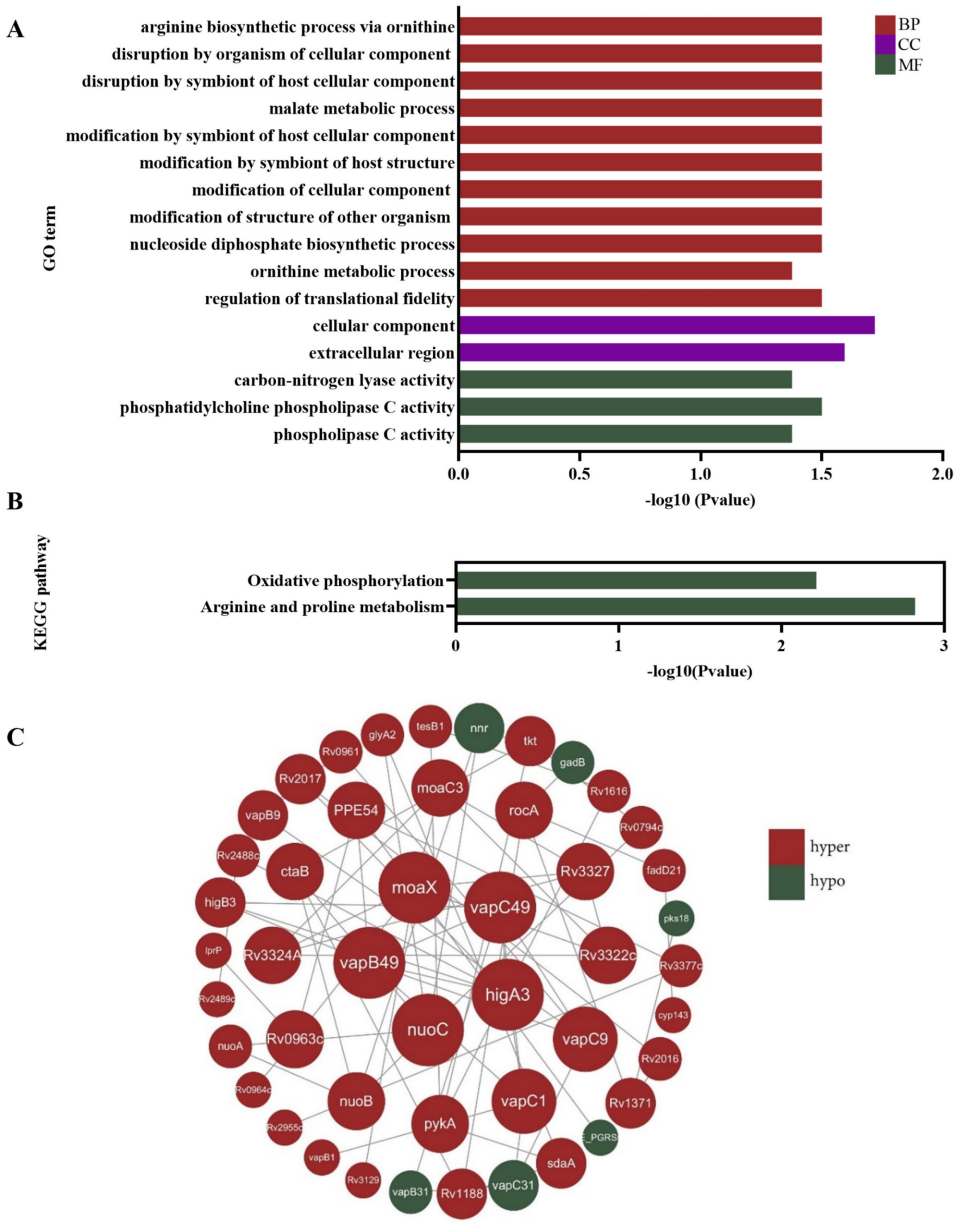
Functional analysis of DNA methylation sequencing results in multidrug-resistant *Mycobacterium tuberculosis*. **(A)** GO analysis revealed that hypermethylated genes were mainly enriched in the arginine biosynthetic process. The GO is organized in three aspects, biological process (BP), cellular component (CC) and molecular function (MF). (**B**) The KEGG pathway functional enrichment analysis showed that hypermethylated genes were linked to arginine metabolism. (**C**) The PPI network of differentially methylated genes showed the top five key genes with the highest degrees were *moaX*, *vapC49*, *vapB49*, *highA3* and *nuoC*. Red nodes stand for differentially hypermethylated genes, while green nodes stand for differentially hypomethylated genes.

Subsequently, the screened differentially hypermethylated genes were analyzed by GO and KEGG pathway functional enrichment analysis. The GO is organized in three aspects, biological process (BP), cellular component (CC) and molecular function (MF). BP enrichment analysis revealed that hypermethylated genes were mainly enriched in the arginine biosynthetic process, disruption by organism of cellular component, disruption by symbiont of host cellular component and malate metabolic process; cell component enrichment analysis indicated that hypermethylated genes were correlated with cellular component and extracellular region; for molecular function, hypermethylated genes were enriched mainly in carbon-nitrogen lyase activity, phosphatidylcholine phospholipase C activity and phospholipase C activity (**Figure 3A**). The KEGG pathway analysis showed that hypermethylated genes were linked to oxidative phosphorylation and arginine and proline metabolism (**Figure 3B**). The STRING database was applied for the construction of the PPI network, hub genes were identified using Cytoscape software. The PPI network for differentially methylated genes was illustrated in **Figure 3C**, the top five key genes with the highest degrees were *moaX*, *vapC49*, *vapB49*, *highA3* and* nuoC*. Additionally, the *rocA* gene was also identified. Although *rocA* is not one of the five hub genes, it was noteworthy that it is associated with the arginine metabolism pathway. These findings suggested that the differentially hypermethylated genes in multidrug-resistant *Mtb* were mainly involved in the arginine biosynthetic process.

### The qPCR results showed that the rocA level in the PBMCs of spermidine^high^ patients was higher than that in spermidine^low^ patients

**Figure 4:**
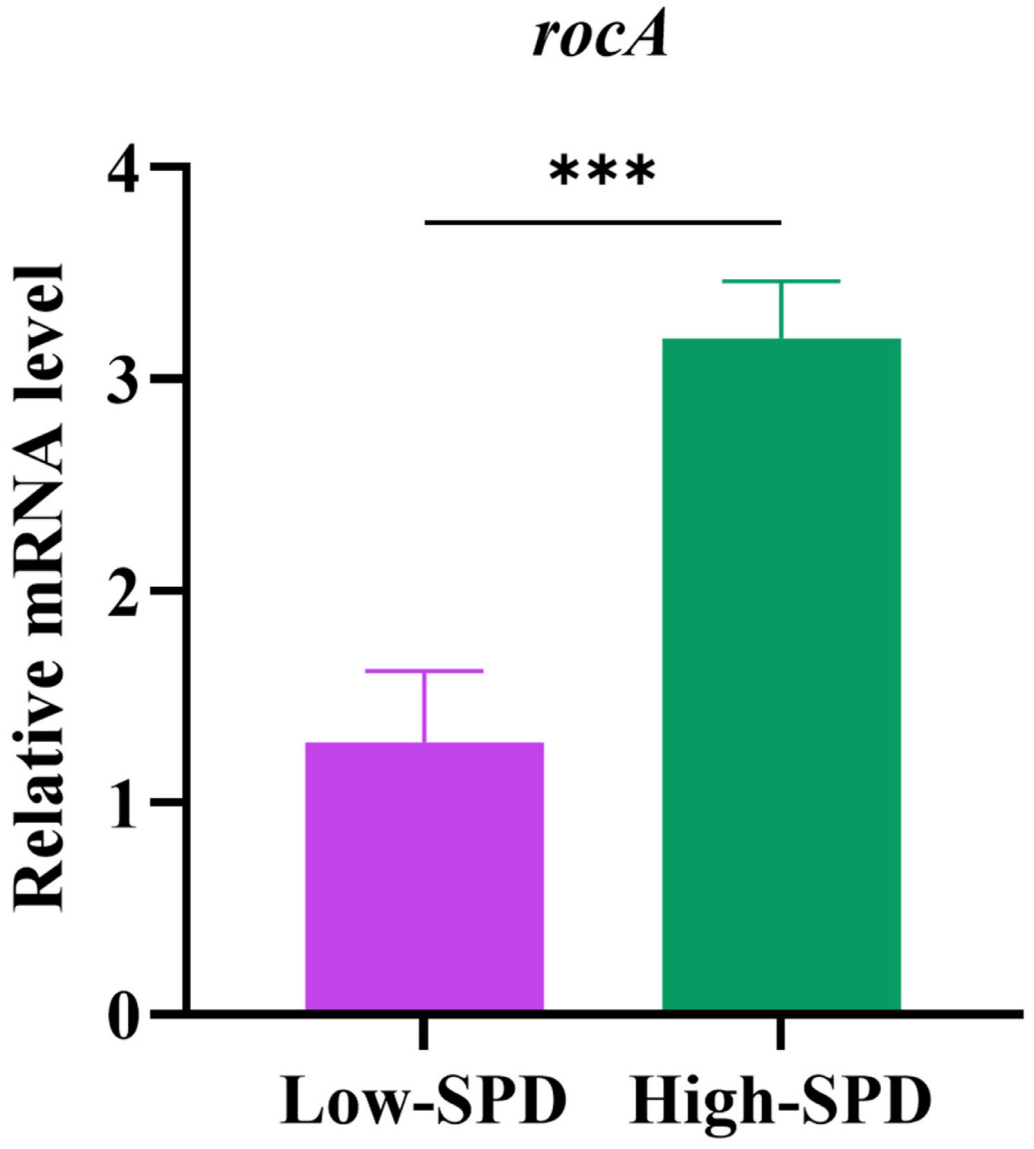
Relative mRNA expression of High-SPD group and Low-SPD group by using qPCR. ***P < 0.0001.

Finally, we assessed the mRNA levels of the *rocA* gene in PBMCs from MDR-TB patients. Based on the levels of SPD in peripheral blood, the patients were categorized into a High-SPD group and a Low-SPD group. The results of qPCR suggested that expression level of *rocA* gene in the High-SPD group was higher than that in the Low-SPD group (**Figure 4**).

## Discussion

The biosynthesis of spermine and other polyamines, is catalyzed by three main enzymes: spermine oxidase (SMOX), ornithine decarboxylase 1 (ODC1), and spermidine synthase (SRM). SMOX catalyzes the conversion of spermine into spermidine, and is a source of cancer-related reactive oxygen species (ROS) and is up-regulated in a wide range of cancers, including colorectal cancer (CRC), non-small cell lung cancer (NSCLC), and gastric cancer 31. Kim *et al.* analyzed 350 cases of colorectal cancer tissues by qPCR, WB, and immunohistochemistry and found that SMOX was overexpressed in both the tissues and clinical samples. In addition, the knockdown of SMOX could inhibit the proliferation of CRC cells, which revealed its oncogenic function 32. SMOX was also found to be highly expressed in NSCLC tissues, and survival curves revealed that patients with lower levels of SMOX had a higher survival rate, suggesting its potential as a predictive target for NSCLC 33. Mice deficient in SMOX showed dramatically reduced levels of gastric spermidine and *H. pylori*-induced inflammation, suggesting that the SMOX gene is associated with an increased risk of human gastric cancer 34. ODC1, an oncogene involved in polyamine biosynthesis, is associated with poor prognosis when up-regulated in various cancers 35. SRM is essential for the production of the genotoxic metabolite colibactin by *Escherichia coli*, and colibactin has been directly implicated in the development of colorectal cancer 36.

Several studies have highlighted the important role of polyamines in the development of several diseases. For instance, supplementation with exogenous SPM has been shown to reverse testicular injury and dysfunction in mice caused by triptolide, with the underlying mechanism involving the upregulation of heat shock proteins 70s (HSP70s) to exert a protective effect 37. Supplementation with SPD has been found to enhance mitophagy activity and mitochondrial function, promoting oocyte maturation and early embryonic development in aged mice 38. Among various kidney injury models, a decrease expression of enzymes for polyamine synthesis and an increase expression of polyamine degrading enzymes was observed 39. Elevated levels of polyamine have also been implicated in the development of Alzheimer's disease 40. Collectively, these studies suggested a significant association between polyamines and the development of multiple diseases.

The purpose of this study was to reveal the potential role of polyamines in patients with MDR-TB by evaluating the content of polyamines in the samples. Firstly, using the HPLC to evaluate the contents of polyamine metabolites SPD and SPM in the peripheral blood of healthy volunteers (HV) and TB patients. The results suggested that the level of SPD in the peripheral blood of TB patients was higher than that in healthy volunteers, while the level of SPM was decreased. Furthermore, 60 TB patients were subdivided into 29 MDR-TB patients and 31 DS-TB patients based on the drug sensitivity results, allowing for an investigation of the mechanisms involving SPD and SPM in multidrug-resistant tuberculosis. Analysis of the levels of SPD and SPM in the peripheral blood revealed that both metabolites were elevated in the MDR-TB patients compared to DS-TB patients. In this study, WB was used to analyze the levels of SMOX, ODC1 and SRM in peripheral blood PBMC samples from HV and TB patients. The results showed that TB patients exhibited elevated protein expression of SMOX, ODC1 and SRM, as well as elevated levels of SPD, which revealed the function of polyamines in the TB disease. Subsequent semi-quantitative analysis of SMOX, ODC1 and SRM levels in the peripheral blood of DS-TB patients and MDR-TB patients using WB and qPCR revealed enhanced expression of SMOX and ODC1 in MDR-TB patients, and the results of the study were in agreement with the conclusions of the reported literature, which indicates that SMOX, ODC1 and SRM, the three key enzymes involved in polyamine metabolism, exhibited elevated levels in a variety of diseases 31-36.

SSAT is a key enzyme involved in polyamine catabolism and plays an important role in the maintenance of polyamine metabolic homeostasis *in vivo*. Under normal circumstances, the SSAT content in the cells is extremely low; however, SSAT expression levels increase rapidly under the high polyamine levels, polyamine analog treatment and multiple pathological stimuli, as the half-life of SSAT is greatly prolonged by the binding of polyamines or polyamine analogs to SSAT. The activation of HIF-1α enhances the bactericidal effect of macrophages against *Mycobacterium tuberculosis* 41. HIF-1α signaling in Müller neuroglia under hypoxic conditions regulates the production of SMOX, which plays a role in the production of oxidative stress inducers 42. The activation of AMPK and mtROS leads to an upregulation of HIF1-α, which is essential for the expression of anti-inflammatory genes as well as the induction of autophagy 43. Nitric oxide synthase (NOS) consists of three isoforms: nNOS (neuronal-type NOS), iNOS (inducible NOS), and eNOS (endothelial-type NOS). In this study, we analyzed the levels of SSAT, HIF-1α and NOS by ELISA, and the results suggested that SSAT and HIF-1α levels were elevated in the peripheral blood of MDR-TB patients, while there was no significant difference in the level of NOS compared to that of DS-TB patients.

The dysregulation of DNA methylation is closely associated with the development of cancer 44, and an overall reduction in the level of 5-methylcytosine modification has been found for the first time in cancer 45. Additionally, while an overall hypomethylation was present in cancer cells, certain localized regions, such as CpG islands, exhibited elevated levels of methylation. This phenomenon may be attributed to lncRNA-mediated DNA methylation 46. DNA methylation is essential for the normal development and function of the human brain, such as the proliferation and differentiation of neural stem cells, synaptic plasticity, neuronal repair, and learning and memory 47. Furthermore, DNA methylation takes an important role in the development and aging process of the retina and may provide a new biomarker for the prediction, diagnosis and prognosis of both diabetic retinopathy and age-related macular degeneration 48. Recently, most methylation studies on drug-resistant* Mtb* have been conducted. The bacterial methylome has been linked to survival under antibiotic stress 49, suggesting that DNA methylation may play significant roles in antibiotic susceptibility. Hu *et al.* demonstrated that mycobacterial HsdM, an adenine methyltransferase, changed the transcription levels of its corresponding substrates by methylating them, thereby reducing the susceptibility of mycobacteria to isoniazid 50. Chen *et al.* investigated genome-wide DNA methylation and transcriptome changes in rifampicin or isoniazid-resistant* Mtb*. Their findings indicated that methylation-mediated regulatory pathways may contribute to the mechanisms of antibiotic resistance in *Mtb* H37Rv 51. Wu *et al.* employed genome-wide methylation, transcriptome, and proteome analyses to elucidate the associations between specific genes and streptomycin resistance in *Mtb* H37Rv. The results revealed that methylation-related genes, such as *coaE*, *fadE5*, and *mprA*, might significantly contribute to the understanding of streptomycin-resistant *Mtb* 52. Consequently, our study investigated the role of DNA methylation in multidrug-resistant *Mtb*. Sputum samples were collected from three patients with drug-sensitive tuberculosis and three patients with multidrug-resistant tuberculosis. DNA was extracted from *Mycobacterium tuberculosis* for methylation sequencing analysis. Subsequently, we analyzed the screened hypermethylated genes by GO and KEGG pathway functional enrichment analyses. GO analysis indicated that hypermethylated genes predominantly participated in the arginine biosynthetic process. Furthermore, KEGG pathway analysis revealed that hypermethylated genes were mainly enriched in arginine and proline metabolism. Moreover, five hub genes, including *moaX*, *vapC49*, *vapB49*, *higA3* and *nuoC*, were identified from PPI network. Although *rocA* gene is not among these five hub genes, it was noteworthy that *rocA* is associated with the arginine metabolism pathway. In conclusion, polyamine metabolites were increased in peripheral blood of MDR-TB patients. The differentially hypermethylated genes in multidrug-resistant *Mtb* are involved in the arginine biosynthetic process, the differentially methylated genes may play an important biological role in multidrug resistance of *Mtb*.

## Figures and Tables

**Table 1:** Basic clinical data of the TB and HV groups

	HV	TB	
		MDR	DS
N	48	29	31
Age	31.920±9.435	38.900±12.930	38.260±14.010
Male/Female	34/16	16/13	17/14
WBC (10^9^/L)	6.115±1.871	5.496±1.327	6.013±3.512
LYMPH (10^9^/L)	1.888±0.563	1.609±0.453	1.563±0.443
MONO (10^9^/L)	0.369±0.130	0.379±0.168	0.442±0.203
NEUT (10^9^/L)	3.650±1.442	3.323±1.164	3.880±3.245
EO (10^9^/L)	0.175±0.345	0.146±0.134	0.110±0.086
BASO (10^9^/L)	0.035±0.022	0.050±0.040	0.061±0.061
LYMPH%	31.732±6.353	30.200±9.275	29.952±11.035
MONO%	6.180±1.658	6.810±2.244	7.600±1.900
NEUT%	58.880±8.381	59.417±10.305	59.052±11.777
EO%	2.636±3.899 0.572±0.318	2.679±2.411	2.342±1.710
BASO%		0.893±0.724	0.965±0.698

WBC, white blood cells; LYMPH, lymphocytes; MONO, monocytes; NEUT, neutrophils; EO, eosinophils; BASO, basophils.

**Table 2:** Gradient elution program table

Time (min)	ultrapure water (%)	Acetonitrile (%)
0	40	60
5	20	80
14	5	95
18	40	60
20	40	60

**Table 3:** qPCR primer sequence

Gene	Sequence (5´-3´)
*SMOX*	CGGATGACCCTCTCAGTCG
	GCGTGTCCAAGTTTCACACT
*ODC1*	GCCATCGTGAAGACCCTTG
	GGCAATCCGCAAAACCAACTT
*SRM*	GTGGTGGCCTATGCCTACTG
+	CTCCTGGAAGTTCGTGCTCG
*rocA*	GCGAATACGACGACAGCGAAG
	ACAGCAGCGGACCGAAGTAC
*GAPDH*	GGAGCGAGATCCCTCCAAAAT
	GGCTGTTGTCATACTTCTCATGG
